# Web-Based Computational Chemistry Education with CHARMMing III: Reduction Potentials of Electron Transfer Proteins

**DOI:** 10.1371/journal.pcbi.1003739

**Published:** 2014-07-24

**Authors:** B. Scott Perrin, Benjamin T. Miller, Vinushka Schalk, H. Lee Woodcock, Bernard R. Brooks, Toshiko Ichiye

**Affiliations:** 1 Laboratory of Computational Biology, National Heart, Lung, and Blood Institute, National Institutes of Health, Bethesda, Maryland, United States of America; 2 Department of Natural Sciences, New College of Florida, Sarasota, Florida, United States of America; 3 Department of Chemistry, University of South Florida, Tampa, Florida, United States of America; 4 Department of Chemistry, Georgetown University, Washington, D.C., United States of America; University of Wisconsin-Madison, United States of America

## Abstract

A module for fast determination of reduction potentials, *E°*, of redox-active proteins has been implemented in the CHARMM INterface and Graphics (CHARMMing) web portal (www.charmming.org). The free energy of reduction, which is proportional to *E°*, is composed of an intrinsic contribution due to the redox site and an environmental contribution due to the protein and solvent. Here, the intrinsic contribution is selected from a library of pre-calculated density functional theory values for each type of redox site and redox couple, while the environmental contribution is calculated from a crystal structure of the protein using Poisson-Boltzmann continuum electrostatics. An accompanying lesson demonstrates a calculation of *E°*. In this lesson, an ionizable residue in a [4Fe-4S]-protein that causes a pH-dependent *E°* is identified, and the *E°* of a mutant that would test the identification is predicted. This demonstration is valuable to both computational chemistry students and researchers interested in predicting sequence determinants of *E°* for mutagenesis.

## Introduction

In biological systems, oxidation and reduction reactions, in which molecules donate and accept electrons, control the flow of chemical energy necessary for life [1]. The ability for a molecule to accept an electron (i.e., to be reduced) is quantified by its reduction potential, *E°*. High-energy processes such as photosynthesis and metabolism utilize electron transfer chains, which consist mainly of redox sites in proteins, to transfer electrons efficiently. In an electron transfer chain, the transfer from the initial to final species should be favorable, implying that the *E°* of the initial species should be higher than the *E°* of the final species. In addition, each redox site along the chain generally has a lower *E°* than the preceding one, or at least not so much higher that the electron becomes trapped. Thus, how the protein environment controls the *E°* of its redox site could aid in identifying malfunctions in diseased electron transfer chains and in designing novel redox proteins.

The history of theoretical approaches to predict the *E°* for redox active proteins is long [2]–[12]. Recently, a combination of density functional theory (DFT) and Poisson-Boltzmann (PB) continuum electrostatics calculations, referred to as the DFT+PB approach, has been proposed for calculating the *E°* of a protein versus the standard hydrogen electrode (SHE) from its crystal structure [12], which gives results in excellent agreement with experiment for multiple [4Fe-4S]-proteins [11]. In addition, this approach has been used in methods to analyze *E°* of proteins, such as in identification of sequence determinants of differences in the *E°* between proteins [13] and of ionizable residues that cause pH-dependence of *E°* in a given protein [14].

The *E°* of a redox-active protein is a function of the free energy of the reduction reaction for the protein. The two important contributions are the free energy of the redox site, Δ*G*_in_, which is a function of the type of redox site, and the free energy of the environment of the redox site, Δ*G*_out_, which is a function of the nature of the protein plus the solvent. In terms of these contributions, *E°* is given by
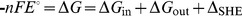
(1)in which *n* is the number of electrons transferred, *F* is the Faraday constant, and Δ*G*_SHE_ is the free energy of an electron in the SHE. Note that *E°* generally refers to *E°*(reduction), the reduction potential; the oxidation potential is simply related to the reduction potential by *E°*(oxidation) = −*E°*(reduction). In the DFT+PB method, Δ*G*_in_ is calculated using DFT and Δ*G*_out_ is calculated using PB. While Δ*G*_in_ is computationally intensive, especially because the common redox sites contain transition metals, the same value can be used for chemically similar redox sites undergoing the same redox couple, so that a library of Δ*G*_in_ values for each type of redox site has been developed. On the other hand, Δ*G*_out_ is based on the coordinates of the protein and so must be calculated separately for each protein. However, it is much less computationally expensive to calculate Δ*G*_out_ and can be performed on a workstation in a matter of minutes. Thus, *E°* can be calculated quickly for a protein with a redox site in the library and a crystal structure in the Protein Data Bank (PDB) [15], making this an attractive addition to an online web server to complement more computationally intensive methods, such as molecular dynamics simulations.

CHARMM INterface and Graphics [16] (www.charmming.org) is a public domain, web-based tool to set up calculations of biological molecules. Since a primary use of this web interface is envisioned as a learning tool for setting up various calculations in Chemistry at HARvard Macromolecular Mechanics (CHARMM) [17], it provides a set of lessons with step-by-step instructions for uploading a protein structure from the PDB; minimizing, solvating, and neutralizing the protein; and initiating a molecular dynamics simulation. While time on the CHARMMing server is limited, all files created in CHARMMing can be downloaded and restarted on another computer with CHARMM, which allows users to set up calculations in CHARMMing and complete them on their own computers.

The usefulness of the redox module is enhanced by the new CHARMMing mutation protocol, which allows sequence determinants of the *E*° of a protein to be identified and tested computationally. Sequence determinants are differences in amino acid sequence, which are generally near the redox site, that give rise to differences in the electrostatic potential at the redox site. For example, a combination of sequence analysis and energy calculations have identified a 0.05 V shift in the *E°* for rubredoxin, depending on whether a certain residue is either an alanine or valine, since the larger valine side chain shifts the polar groups of the backbone away from the redox site [6]. Similarly, a 0.1 V shift in the *E°* for [4Fe-4S]-containing ferredoxins is associated with a cysteine versus an alanine because of changes in both the polarity of the side chain and position of the backbone [10]. Both of these cases have been verified experimentally [8], [18]. Lastly, a 0.025 V shift in *E°* with pH due to change in the protonation state of the single histidine in *Chromatium vinosum* HiPIP has been calculated, which is also in good agreement with experiment [14], [19]. All of these examples involve a change in Δ*G*_out_ that can be explored by mutating residues of interest in a redox protein in CHARMMing.

Here, we discuss the implementation of *E°* calculations in CHARMMing and how these calculations can be used to analyze the environmental contribution to *E°* of a protein. The procedure and associated CHARMM files are briefly discussed. In addition, a lesson for the redox module provided in CHARMMing is described here, which is useful for both students learning computational chemistry and researchers interested in predicting sequence determinants of *E°* for mutagenesis.

## Calculation of the Reduction Potential in CHARMMing

**Figure 1:**
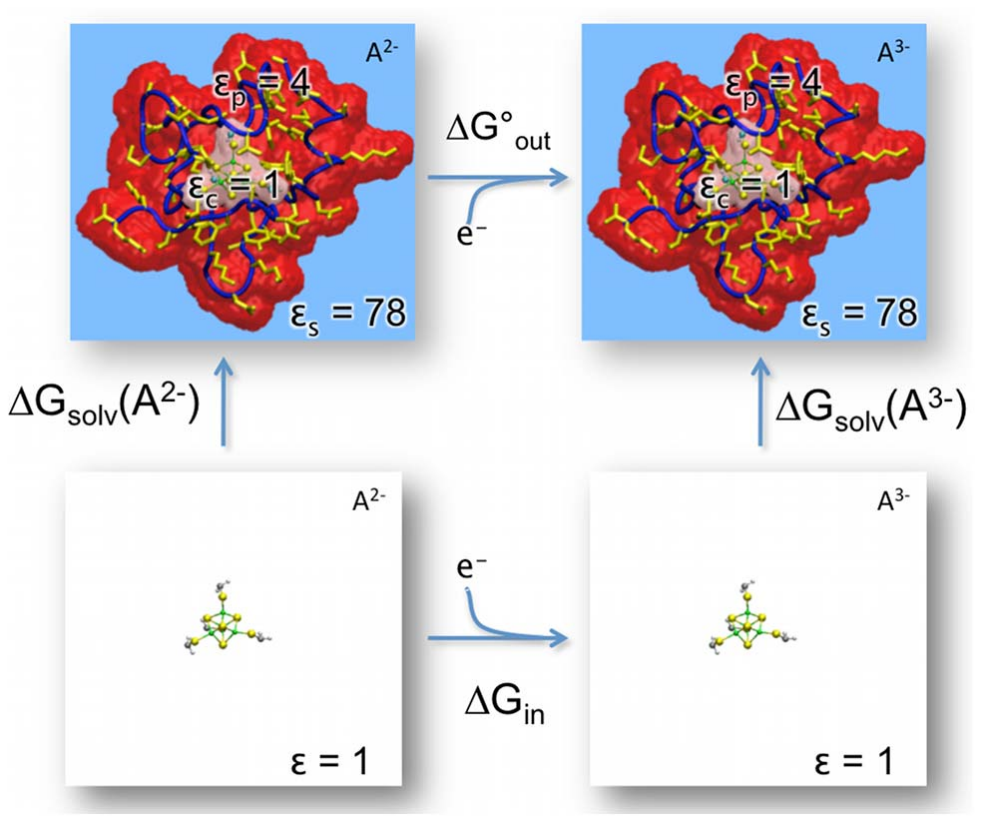
Thermodynamic cycle for calculating the absolute free energy of reduction (Δ*G*) for an iron-sulfur protein containing the redox site A. The environmental contribution Δ*G*_out_ = Δ*G*_solv_(A^3−^)−Δ*G*_solv_(A^2−^).

The *E°* in CHARMMing involves determining Δ*G*_out_ from a series of PB calculations using the program APBS [20] through the CHARMM iAPBS interface [21], while Δ*G*_in_ is provided via a redox parameter library. Since Δ*G*_out_ = Δ*G*_solv_(A*^n^* ^−1^)−Δ_solv_ *G*(A*^n^*), where *n* is the initial oxidation state of the redox site A (Figure 1), the “solvation” energy Δ*G*_solv_ of the redox site in both oxidation states must be calculated. Like a typical definition of solvation energy, Δ*G*_solv_(A) is the difference in free energy between the entire system and a reference system, which consists of A in vacuum. However, unlike a typical definition, A is only the redox site and the solvation is by the rest of the protein plus its surrounding environment; thus, the atoms of the redox site and those of the rest of the protein must be defined into separate segments. Since an oxidation/reduction reaction in the type of redox sites found in biology generally involves changes in electron density spread over multiple atoms, a reasonable definition for the redox site is to include heteroatoms plus any protein side chains that are directly bonded to a heteroatom. This also means that the oxidation state of the redox site is defined by its partial charges, which are also provided in the redox parameter library, along with atomic radii. Since partial charges and atomic radii for all atoms are required for a PB calculation, the rest of the proteins are obtained from the CHARMM36 parameters [22].

**Figure 2:**
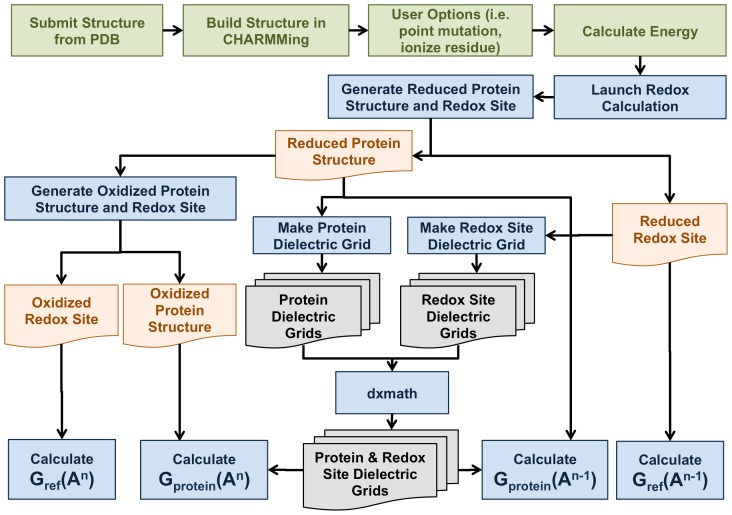
Flow chart of redox calculations in CHARMMing. Green boxes represent the CHARMMing steps before performing the redox calculation. Blue boxes represent processes within the redox module. Orange files are CHARMM PSF and CRD files, while gray files are the dielectric grids.

Operationally, CHARMMing builds four structures based on this information, the oxidized and reduced states of the entire system, and the oxidized and reduced states of the reference system. CHARMMing separates the original coordinates into three segments: (1) the amino acid residues, (2) “good” heteroatoms found in standard topology and parameter files, and (3) “bad” heteroatoms not in standard topology and parameter files, as discussed by Miller et al. [16]. Redox site atoms are considered “bad” heteroatoms. The structure-editor will reassign side chain atoms from the protein to the redox site if necessary. Next, non-unique atoms whose charges change differentially are renamed and the redox site is given a new residue name for each oxidation state. This results in protein structure files (PSF) and coordinate files (CRD) for the reduced redox site in the protein (Reduced Protein Structure in Figure 2) and in vacuum (Reduced Redox Site in Figure 2), as well as oxidized versions of these files (Oxidized Protein Structure and Reduced Redox Site, respectively, in Figure 2).

After setting up the structures, the redox calculations are performed. First, CHARMMing generates dielectric grids for the solvated protein (Protein Dielectric Grids in Figure 2), which contains three redox regions of ε_c_ = 1 for the redox site volume, ε_p_ = 4 for the protein volume, and ε_s_ = 78 for the solvent, and for the reference state (Redox Site Grids in Figure 2), which contains one redox region of ε_c_ = ε_p_ = ε_s_ = 1. Then, the PB equation is solved for the reduced states of the entire system and the reference system (two blue boxes on bottom right in Figure 2) and the oxidized states of the entire system and the reference system (two blue boxes on bottom left, respectively, in Figure 2), where the oxidized and reduced states are differentiated by the partial charges of the redox site. From the results, Δ_solv_ *G*(A*^n^*) and Δ_solv_ *G*(A*^n^* ^−1^) are calculated by taking the difference between the protein and reference systems in the appropriate oxidation state, and the final value of *E°* evaluated by Equation 1. For convenience, Table S1 lists the names of files created with the module with a brief description.

## Graphical Simulation Setup Features

**Figure 3:**
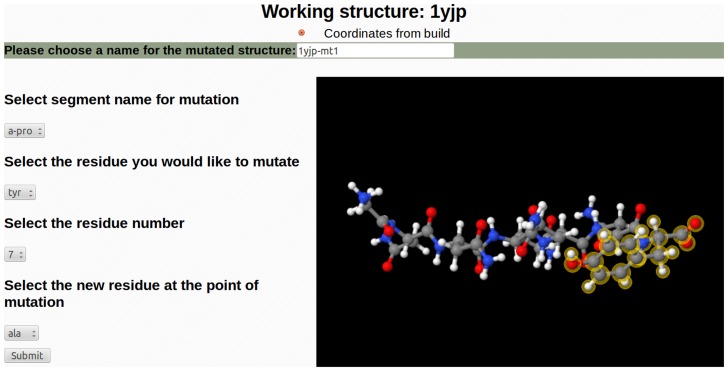
Example of the graphic interface for making point mutations in CHARMMing.

Computational single-site mutation is a way to quickly test the effects of the single residue substitution, especially as a prelude to experimental mutation. For instance, it is a means of testing the effects on *E°* of mutations being considered to verify the importance of residues identified as being large contributors to the *E°* of a protein. The graphical simulation in CHARMMing allows the user to easily create a new working structure by mutating a single amino acid of the existing working structure using JSmol (Figure 3) [23]. The area highlighted in the JSmol window on the mutations page changes depending on which residue is selected, which allows the user to more easily see the environment around the residue that is selected for mutation. The replacement also entails slight optimization of the structure of the mutant residue and protein within 10 Å.

## Redox Lesson

CHARMMing includes a lesson that guides a user through a redox calculation involving the histidine responsible for the pH-dependence of the *E°* in *Chromatium vinosum* (*Cv*) HiPIP (PDB ID: 1CKU) [24], which contains a [4Fe-4S] redox site that undergoes a 1-/2- redox couple, based on a previously reported calculation [14]. *Cv* HiPIP has only one histidine at residue 42, which is thought to be responsible for the pH-dependence [19]. The lesson demonstrates how to accomplish several tasks:

*Step 1.* How to upload coordinates of the protein from the PBD and build a structure.
*Step 2.* How to calculate *E°* for a protein. Titratable residues are assumed to have standard charges at pH 7.
*Step 3.* How to determine the residue(s) that causes pH dependence of *E°*. The protonation state of titratable residue is altered to create a new modified protein. The difference between *E°* for the modified protein and *E°* from Step 2 gives the change expected if the *E°* at the new pH is due to that residue. Multiple residues should be modified if they are all expected to change at the new pH.
*Step 4*. How to determine the contribution of a residue to *E°*. A charge knock-out, which sets the partial charges for specified atoms to zero, is performed on the residue of interest to give a charge knock-out protein. The difference between *E°* from the previous step (in this case, step 3) and *E°* for the charge knock-out protein gives the contribution of the residue (in this case, the protonated histidine). Residues with absolute contributions greater than 0.03 V can be considered as sequence determinants of *E°*.
*Step 5.* How to determine the effects of a site-specific or point mutation. The residue of interest is mutated to a target residue to create a second modified protein. The difference between *E°* for the modified protein and *E°* from Step 2 (or 3) gives the change from wild-type expected upon mutation at pH 7 (or low pH).

Successful completion of the lesson will give the prediction that protonation of the histidine at low pH should increase *E°* by 50 mV relative to neutral pH while a mutation to an alanine will slightly decrease *E°* by 30 mV relative to the wild-type at neutral pH. The lack of ionization will mean the wild-type at low pH will be 90 mV higher than the mutant.

### Step 1. Upload a [4Fe-4S]-containing protein

**Figure 4:**
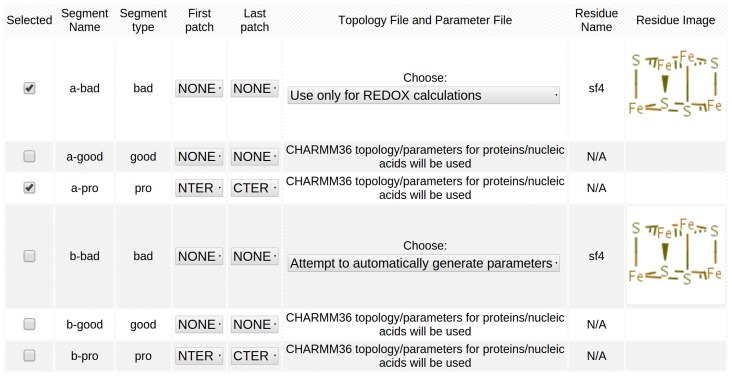
Example of Structure Editing module setup for iron-sulfur containing proteins.

Navigate to the “Submit Structure” page on from the main menu. Select “Retrieve a PDB using a PDB ID” and enter the PDB.org ID “1CKU” into the text box, select Lesson 6 from the drop-down menu for “What Lesson is this structure associated with?” and submit the structure. CHARMMing will redirect the page to the “Build/Select Working Structure” page. Here, an arbitrary name can be provided for the structure and the atoms from the PDB are selected. Since “1CKU” contains two independent proteins in the asymmetric unit, the atoms of one protein are selected under “Choose Segments and Patching” as the “a-pro” segment, containing all protein atoms for monomer A, and the “a-bad” segment, containing the redox site atoms for monomer A. To load the redox site parameters, select “Use only for REDOX calculations” from the drop-down menu under “Topology File and Parameter File” for “a-bad” (Figure 4). The segment should appear as long as the redox site is in the redox parameter library (currently, only cysteine-ligated [4Fe-4S] cofactors of the iron-sulfur proteins, named FS4 and SF4 in the PDB, are supported). Submit the page, and a message should appear indicating the protein structure was successfully built. In addition, CHARMMing currently requires an energy calculation prior to the redox calculation. Select “Energy” under the “Calculations” header in the main menu. Choose “Calculate Energy” using the default values, and the calculation will run in the background. Once the status for the energy calculation says “Done,” navigate back to the “Energy” page and a selection of CHARMM output should be on the screen, stating that the structure has a total potential energy of approximately −82 kcal/mol under the CHARMM force field.

### Step 2. Calculate E° for the wild type

**Figure 5:**
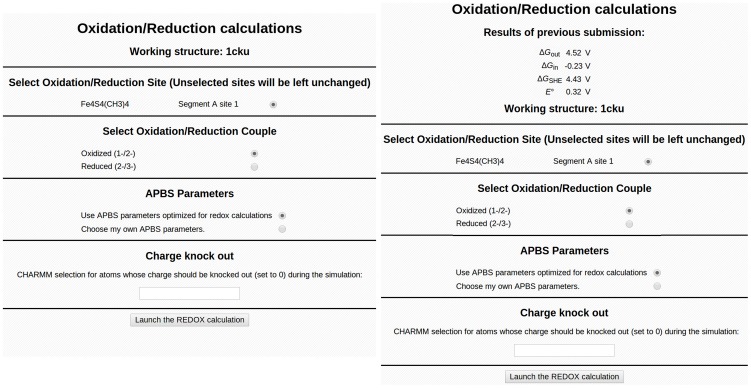
Initial submission form (left) and submission form showing results (right) for the redox module.

Navigate to the Redox page under the “Analysis” header (Figure 5, left). Select the Oxidation/Reduction Site, “Fe4S4(CH3)4 Segment A site 1.” If a protein contains multiple redox sites, only one site can be specified at a time, so the *E°* for each site must be calculated individually. Select the 1-/2- redox couple under “Select Oxidation/Reduction Couple.” In addition, CHARMMing only calculates the *E°* for single electron reduction; for a double reduction, the *E°* must be calculated separately for the addition of each electron. The default values of the PB parameters optimized for [4Fe-4S]-containing proteins [11] should be specified, but advanced users may change many of these. Then select “Launch the REDOX calculation!” When the status of the redox job says “Done,” select “Redox” from the Analysis submenu again to retrieve the calculated *E°* (Figure 5, right), which should be 0.32 V.

### Step 3. Identify the residue that modifies E° by changing its charge state

Return to the “Build/Select Working Structure” page and rebuild a new structure of *Cv* HiPIP with a protonated His 42. Before submitting the page, check “Modify protonation states of titratable residues,” which will give a list of titratable residues. For residue 42 of SEGID “a-pro,” select Protonation State “hsp” from the drop-down menu. Submit the structure, and repeat steps 1 and 2. The new *E°* should be 0.37 V, which means the predicted increase in *E°* due to protonation of His 42 is 0.05 V.

### Step 4. Evaluate the contribution of a residue to E° using charge-knockout

The contribution of a protonated His 42 side chain to the *E°* is determined by turning off the partial charges of that residue. Since the protein structure currently contains a charged histidine after step 3, the structure does not have to be rebuilt. Navigate back to the redox page and repeat step 2, but enter “resid 42” into the “Charge knockout” text box, then select “Launch the REDOX calculation.” The *E°* should now be 0.28 V. The difference between the *E°* from step 3 (0.37 V) and the one provided by the knock out (0.28 V) will give the contribution of a protonated His 42 to the *E°* (0.09 V).

### Step 5. Predict E° for Cv HiPIP with a point mutation

To model a mutation of *Cv* HiPIP, navigate to the “Modify Structure by Point-Mutation” page. Select the residue HSP 42 and replace it with an alanine (ALA). The graphical representation will show the position of the mutation on a three-dimensional model of *Cv* HiPIP. Return to the redox module and calculate the *E°* for the mutant. The new *E°* should be 0.29 V.

## Discussions and Conclusions

The redox module in CHARMMing is both a teaching tool for a graduate or advanced undergraduate curriculum and a research tool for structural biologists. As a teaching tool, the fast *E°* calculations allow an instructor to demonstrate a fundamental computational chemistry research application within the timeframe of a lecture or homework assignment, while as a research tool, the fast *E°* calculations allow a researcher to investigate the contributions of multiple residues to *E°*, as well as the effects of many different mutations on *E*°, particularly as a prelude to time-consuming experiments. The accompanying lesson provides examples for how to analyze *E*° using CHARMMing.

In this CHARMMing lesson, the change in *E°* for a metalloprotein (*Cv*HiPIP) associated with titration of an ionizable residue (His42) is demonstrated. The measured *E°* is 0.355 V at high pH and 0.377 V at low pH [19], which agree with the calculated values of 0.32 V for neutral histidine and 0.37 V for the charged histidine. The H42Q mutant has an experimentally determined *E°* of 0.357 V compared to 0.29 V calculated with CHARMMing. This difference is likely due to the conformation of the glutamine built with the CHARMMing mutation protocol. A better value may be obtained by averaging over several confirmations of the mutated residue.

The error in the calculated *E°* is generally around 0.05 V and is dependent on parameters for the redox calculation and the resolution of the crystal structure. The PB parameters in the online lesson are optimized for performance on the CHARMMing servers by using a calculation grid with a 0.4-Å spacing between grid points. More accurate *E°* can be calculated within CHARMMing by selecting “Choose my own APBS parameters” in the redox calculation page (Figure 5, left). For best results, a grid spacing of 0.2 Å is suggested. Accuracy in *E°* for a HiPIP protein structure as a function of grid spacing is discussed in reference [12].

## Supporting Information

Table S1 Names of files produced by the CHARMMing Redox Module with a brief description.
(PDF)
