# Supplementary material for: Web-Based Computational Chemistry Education with CHARMMing III: Reduction Potentials of Electron Transfer Proteins
Source: PLoS Comput Biol. 2014 Jul 24;10(7):e1003739. doi: 10.1371/journal.pcbi.1003739 (PMC4110074; doi:10.1371/journal.pcbi.1003739)
Supplement: Table S1 — Names of files produced by the CHARMMing Redox Module with a brief description. (PDF) [file pcbi.1003739.s001.pdf]

Supporting Information for Web-based computational chemistry lessons in CHARMMing III: Reduction potentials of electron transfer proteins

Table S1. Names of files produced by the CHARMMing Redox Module with a brief description.

Filename	Description
**Redox Module Files**	
redox-1cku-1-a-pro_r.pdb	Reduced protein PDB
redox-1cku-1-a-bad_r.pdb	Reduced redox site PDB
redox-1cku-1-a-pro_o.pdb	Oxidized protein PBD
redox-1cku-1-a-bad_o.pdb	Oxidized redox site PDB
redox-1cku-1-build-redall.inp/out	I/O for building reduced redox protein
redox-1cku-1-redall.crd/psf	Reduced protein CRD and PSF
redox-1cku-1-build-redsite.inp/out	I/O for building reduced redox site
redox-1cku-1-redsite.crd/psf	Reduced redox site CRD and PSF
redox-1cku-1-build-oxiall.inp/out	I/O for building oxidized redox protein
redox-1cku-1-oxisite.crd/psf	Oxidized protein CRD and PSF
redox-1cku-1-build-oxisite.inp/out	I/O for building oxidized redox site
redox-1cku-1-oxisite.crd/psf	Oxidized redox site CRD and PSF
redox-1cku-1-griddim.str	Stream file containing parameters for PB calculation
redox-1cku-1-redpot.inp/out	I/O for calculating reduced protein potential
redox-1cku-1-redpotref.inp/out	I/O for calculating reduced redox site potential
redox-1cku-1-oxipot.inp/out	I/O for calculating oxidized protein potential
redox-1cku-1-oxipotref.inp/out	I/O for calculating oxidized redox site potential
redox-1cku-1-redpot.txt	Reduced protein potential in kcal/mol
redox-1cku-1-redpotref.txt	Reduced redox site potential in kcal/mol
redox-1cku-1-oxipot.txt	Oxidized protein potential in kcal/mol
redox-1cku-1-oxipotref.txt	Oxidized redox site potential in kcal/mol
**Dielectric Grid Files**	
redox-1cku-1-mkgrid-site.inp/out	I/O for writing redox site only dielectric grids
ref.x/y/z	Redox site only dielectric grids
redox-1cku-1-mkgrid-full.inp/out	I/O for writing protein only dielectric grids
pro.x/y/z	Protein only dielectric grids
redox-1cku-1-combinegrid-site.inp/out	I/O for combining protein and redox site grids
combo.x/y/z	DXMath input files
iapbs-dielx/y/z.dx	Dielectric grids for protein and redox site regions
